# Barriers Interfere With Wide Usage of NOAC for Prevention of Thromboembolic Events Among Doctors in Sudan: A Cross-Sectional Survey February 2023

**DOI:** 10.1155/crp/5028924

**Published:** 2025-05-15

**Authors:** Elaf Sabri Khalil, Asmaa Elfatih Hussein Omer, Wadaha Mohamed Nouh Mohamed, Mustafa Sabir Abakar Awad

**Affiliations:** ^1^Department of Medicine, Al-Neelain University Faculty of Medicine, Khartoum, Sudan; ^2^Department of Community Medicine, Al-Neelain University Faculty of Medicine, Khartoum 11121, Sudan

## Abstract

**Background:** Ischemic heart disease and stroke kill 25% of people worldwide. Vitamin K antagonist (warfarin) is the most widely used oral anticoagulant. Although affordable and effective, its usage is limited in many patients due to anticoagulation level variability and other factors, its alternatives include new nonvitamin K antagonist oral anticoagulants (NOACs). The study aims to investigate NOAC usage barriers.

**Methods:** This is an observational, cross-sectional study, involved 144 doctors from different specialties and different medical degrees in Khartoum state, the data were collected by an author designed close-ended questionnaire. Data were entered, cleared and analyzed using Statistical Package for Social Sciences (SPSS) V25.0 software.

**Results:** Medicine was most common (45.8%) among 144 medical department participants. The most prevalent medical degrees were registrars (25%) and doctors (25%). Specialists (22.9%), then house officers (15.3%). Over half (51.4%) had worked less than 5 years. 50% did not know about the 2021 DOACs guideline. 60.4% claimed DOACs' unavailability inhibits prescription. The lack of a multidisciplinary team approach hinders DOACs prescription, said 70.2%.

**Conclusion:** Sudanese clinicians' hurdles to using NOAC for thromboembolic episodes were explored. Lack of a reversal agent and multidisciplinary team approach hinder DOAC prescription. Lack of information about international guidelines, since most participant's preferred specialized advice or personal experience, and high DOAC costs and inaccessibility and unavailability are other important barriers. Medical practitioners should update guidelines and government insurance plans should include DOACs. Each department should start studies separately.

## 1. Introduction

Thrombosis (VTE) is a prevalent pathological condition that underlies ischemic heart disease, ischemic stroke, and venous thromboembolism. Ischemic heart disease and stroke collectively contribute to almost 25% of global mortality. The utilization of anticoagulant medication has experienced a surge in popularity in recent decades, mostly attributed to its well-documented effectiveness and extensive application across various diseases [1].

Warfarin, an oral anticoagulant, is the most widely utilized vitamin K antagonist globally. Although cost-effective, the use of this treatment is restricted in numerous individuals due to the unpredictable nature of anticoagulation levels, hence heightening the potential for thromboembolic or hemorrhagic consequences. Additionally, it requires frequent blood tests and modifications to the dosage. Nonvitamin K antagonist oral anticoagulants (NOACs), also referred to as Direct OACs (DOACs), are a novel category of medications that are currently being evaluated as potential substitutes for warfarin [2].

Novel OACs (NOACs) exhibit a more focused mechanism of action that specifically targets a single clotting factor. By inhibiting factor Xa and direct thrombin (factor IIa), these medications generate a more reliable and stable anticoagulant effect. This effect is less prone to being influenced by interactions with other drugs or food, and does not require frequent monitoring [1].

The safety and effectiveness of warfarin therapy rely on maintaining the International Normalized Ratio (INR) within the specified target range for the specific indication. Upon initiation of oral anticoagulant therapy, it is necessary to do daily INR monitoring until the INR falls into the therapeutic range for a minimum of two consecutive days. Perturbations in the INR levels of a patient who is otherwise medically stable may arise due to alterations in dietary habits, nonadherence to prescribed treatments, unreported substance abuse, alcohol ingestion, or self-administration of medications. When encountering unexpected values, it is important to additionally consider the possibility of laboratory error. Hemorrhaging is a significant hazard associated with warfarin treatment, and it is strongly linked to INR values [2].

There are multiple reasons that support the use of a switching strategy when there are no randomized comparison studies available to compare switching to NOACs with continuing VKA medication. There is irrefutable evidence that NOACs result in significantly fewer cases of hemorrhagic strokes and cerebral bleeding compared to warfarin. NOACS significantly decreases the likelihood of cerebral bleeding by 52% in comparison to warfarin, with a range of 33%–70%. This advantage is valid for many NOACs and is not influenced by warfarin's duration spent inside the therapeutic range. Furthermore, NOACs exhibit a lower incidence of drug-drug and drug-food interactions in comparison to warfarin. With the exception of patients with mild renal impairment, who should have regular serum creatinine monitoring, NOACs do not require frequent blood tests. The elevated expense of NOACs remains a hindrance to their extensive use, especially in low-income environments [3].

Drug-related issues are well recognised as a major contributor to mortality and a decline in quality of life on a global scale. It has been estimated that between 5% and 10% of hospital admissions can be attributed to drug-related problems, with over half of these cases being preventable. Sudan has a significant population of patients who are prescribed anticoagulant medications, particularly warfarin as the primary choice, along with NOAC. Consequently, enhancing the available treatment alternatives is a key priority [4].

The drawbacks of warfarin, such as the rising mortality rate, declining quality of life from side effects, increased hospitalisations, and the issue of drug interactions with specific foods, highlight the need to thoroughly investigate the potential benefits of NOAC. Understanding the obstacles that hinder the widespread use of NOAC is crucial in order to find solutions and enhance anticoagulation treatment in Sudan.

## 2. Research Methods

### 2.1. Study Design/Setting

An observational, cross-sectional survey, conducted in Khartoum state targeting the three major cardiac centers: Sudan Cardiac Center, AlShaab Teaching Hospital and Ahmed Gasim Hospital. These comprised the largest three cardiac centers in Sudan at the time but due to the unfortunate events of the armed conflict that started at April 2023 they're currently out of service. These centers provide cardiac and cardiac related services to all Sudanese population, they are equipped with all necessary laboratory investigations and catheter related procedures. All of the three centers fall under the national insurance program provided by the government to the people, so all registered patients can benefit from the services. Due to the relatively small sample size that was obtained from the centers an online form of the questionnaire was distributed on multiple social media platforms (Facebook, Telegram and WhatsApp) targeting medical practitioners from different specialties working at different Khartoum state hospitals during the period from October 2022 to April 2023.

### 2.2. Variables

Many variables were considered including doctors related barriers such as lack of knowledge about NOAC due to it novelty in medical practice, fear of dealing with its complications due to absence of reversal agents and the lack of knowledge about the new guideline's recommendations. NOAC related variables as its high price in Sudan, it scarcity in Sudanese market and not being covered by most health insurance plans. Conditions in which NOAC is not preferred over warfarin like mechanical heart valves and rheumatic mitral stenosis (moderate and severe) is a confounding variable that was considered.

### 2.3. Sample Size and Sampling Technique

Through a nonprobability-convenience sampling method, all doctors who met the inclusion criteria and agreed to participate during the study period were included with a total of 144 doctors from different medical departments.

### 2.4. Data Collection

The data were collected using a semistructured, author designed close-ended questionnaires, administered by self-administration via Google form. The questionnaire consisted of four domains assessing general information, barriers related to NOAC, barriers related to health care provider and barriers related to the patient with 35 close ended questions and one open question for any additional comment/barrier. The demographic characteristics of the participants were not obtained as the authors view was to assess the barriers merely on the basis of the clinical background of the participants, although a demographic data would have been useful in comparing different age group, universities, sex and even ethnicities. Information on participant's characteristic like (the department where they works, medical degree, years of experience), medication-related barriers, logistical and financial related barriers, health care providers and system-related barriers were assessed according to WHO recommendations. The questionnaire was applied as a pilot study in a group of 18 physicians to assess for spelling errors, clarity and ambiguous questions. The data was collected by fifth-year undergraduate medical students from Al-Neelain University with a supervision from an internal medicine specialists.

### 2.5. Data Analysis Plan

The collected data were entered to a Microsoft Excel sheet (2016) to be cleared from any duplication and unanswered questions. Then it was analyzed using the statistical package for social sciences (SPSS) V25.0 software. Descriptive statistics including frequency, percentages, the mean, and standard deviation in addition to Chi-square test and regression analysis to compare and correlate different sets of data were applied. A *p* value of 0.05 or less is considered statistically significant.

### 2.6. Ethical Consideration

The research ethical approval was being obtained from the ministry of health and the ethical committee at Al-Neelain University Faculty of Medicine. All participants were provided informed consent. Coding numbers were put on the questionnaire instead of names to ensure confidentiality. Information were collected for research purposes only. The privacy of doctors was considered.

### 2.7. Availability of Data and Materials

The dataset used and/or analyzed during the current study available from the corresponding author on reasonable request.

## 3. Results

The main reason why clinicians abide the utilization of NOACs over VKA was their lack of knowledge about the recent guidelines.

A total of 144 participants were included in this study, the most common department comprised the population was medicine department comprising (45.8%) as in Table 1. The most prevalent medical degree of the total number of doctors involved in this study was registrars (25%) and medical doctors (25%), specialists (22.9%), consultants (11.8%) and then house officers (15.3%), as shown in Table 2.  Table 3 shows the experience level within the participants and the most frequent experience level reported was less than 5 years (51.4%).

Out of all 144 participants, 50.7% were not aware about 2021 new guidelines recommendations regarding DOACs usage. While 49.3% did have knowledge as demonstrated in Table 4.

There was a significant association between the type of the drug prescribed for prevention of thromboembolic events and department as DOACs was mostly prescribed by ER department (40%) heparin by orthopedics department (48.6%) and warfarin was evenly distributed (25%) (*p* < 0.001) as shown in Table 5.

There was a significant association between the first line prescribed for the prevention of thromboembolic events nonvalvular atrial fibrillation. DOACs were prescribed mostly by medicine department (77.1%) and warfarin was also prescribed mostly by medicine department (58.5%) (*p* < 0.001) Table 6.

There was a significant association between the type of drug and department for using it as a first line medication for the prevention of thromboembolic events in patients with atrial fibrillation due to moderate to severe mitral stenosis caused by rheumatic heart disease. DOACs were prescribed mostly by medicine department (63.2%) followed by warfarin (71.9%) also by medicine department (*p* < 0.001) as shown in Table 7.

Table 8 shows association between different variables for instant Medical degree correlated positively with the years of experience (*r* = 0.650) (*p* < 0.001). The level of the rank of medical degree (*r* = −0.219) (*p* < 0.01) (*r* = −0.211) (*p* < 0.05), respectively. The prescription of DOACs positively correlated with the rank of medical degree (*r* = 0.371) (*p* < 0.001). The years of experience inversely correlated with the level of perception about barriers related to doctors (*r* = −0.194) (*p* < 0.05). Perception towards barriers related to doctors, and thoughts related to DOACs inversely correlated with Medical degree rank (*r* = −0.219) (*r* = −0.211) (*p* < 0.001).

Perception about barriers related to doctors inversely correlated with direct oral anticoagulant prescription (*r* = −0.266) (*p* < 0.05).

Perception about barriers related to doctors positively correlated with perception about barriers related to thoughts about the drug (*r* = 0.341) (*p* < 0.001), barriers about the resources (*r* = 0.366) (*p* < 0.001), and barriers related to insufficient reporting (*r* = 0.195) (*p* < 0.05). Perception about barriers related to patient doctor relationship positively correlated with barriers perception related to thoughts about DOACs (*r* = 0.393) (*p* < 0.001).

Perception about barriers related to patient doctor relationship correlated positively with perception of barriers related to thoughts about DOACs (*r* = 0.393) (*p* < 0.001) and to perception of insufficient reporting as a barrier (*r* = 0.176) (*p* < 0.05).

Perception of barriers related to thoughts about DOACs inversely correlated with DOAC prescription (*r* = −0.229) (*p* < 0.001). It positively correlated with perception of insufficient reporting as a barrier (*r* = 0.166) (*p* < 0.05) and resources as a barrier (*r* = 0.284) (*p* < 0.001). As illustrated in Table 8.

## 4. Discussion

The objective of our study was to gather comprehensive and interdisciplinary perspectives from physicians regarding the perceived obstacles to the widespread adoption of DOACs. We intentionally selected individuals from various disciplines and medical degrees, representing diverse sorts of health care delivery zones within Khartoum state. This study had a total of 144 participants, with the majority of the participating doctors being from the medicine department. The most common medical degrees among the doctors participating in this survey were registrars (25%) and medical doctors (25%). Specialists accounted for 22.9% of the total, while consultants made up 11.8%. House officers represented 15.3% of the doctors engaged.

Concerning the obstacles pertaining to doctors, the findings indicate that 85% of them acknowledged the high effectiveness of NOACs. However, only 49.3% of the doctors were aware of the recommendations outlined in the 2021 guidelines. This lack of awareness is not uncommon among doctors, as evidenced by a study conducted by Murphy et al. [5] which focused on their knowledge. 34.8% of respondents concurred with the assertion that patients with renal impairment should abstain from taking NOACs, even though the Murphy study is limited to Irish population and does not constitute a comparable group. Similarly, 34.8% of the medical professionals surveyed held the belief that patients with renal impairments should refrain from using NOACs. Only 17% of the respondents opined that all patients with valvular rheumatic heart disease should avoid NOACs. The responses indicate a significant lack of understanding on the 2021 guidelines among the clinicians participating in the survey. In regards to the preference of doctors for warfarin over NOAC, 34.7% expressed a greater level of comfort with warfarin. Conversely, 68.9% reported being hindered from prescribing NOAC due to their supervisor's preference. This indicates the prevalence of traditional practices over more modern ones, which is unfortunately a common phenomenon among Sudanese medical professionals. They tend to adhere to familiar and proven methods rather than adopting recent updates in practice. This can be attributed to the widely held belief of “if something is effective, there is no need to alter it.”

When it comes to the obstacles of using NOAC in various medical departments, physicians or individuals in the medical field had the lowest average score (12.89) compared to the surgical department, specifically orthopedics (14.83). This suggests that there is a wider variation in the use of NOAC among physicians compared to orthopedic surgeons. This difference can be partially attributed to the fact that physicians rely more on guidelines and updates in their practice, rather than solely relying.

Conversely, the patient-doctor relationship barriers had a lower average score in the surgical department [6] compared to the medicine department (10.21). This may indicates that physicians in the medicine department engage in more detailed discussions about their patients' treatment and management plans than surgeons. This difference can be attributed to the reduced interaction between patients and surgeons, as well as the specific practices of physicians.

The consultants had the lowest mean score (27.29) while the house officers had the highest mean score (32.73) when it came to barriers of thinking on the drug in relation to medical degree. This disparity in scores highlights the knowledge gap between these two groups.

The study found a strong correlation between the type of drug prescribed for preventing thromboembolic events and the departments in which they were prescribed. The emergency department mostly prescribed NOACs (40%), while the orthopedic department mostly prescribed Heparin (48.6%). Warfarin was distributed evenly among departments, with a statistically significant difference (*p* < 0.001). However, it is important to note that NOACs are known to be more effective than LMWH in preventing VTE, as stated by Shakespeare et al. This finding suggests a lack of appropriate knowledge among orthopedic surgeons [6].

We discovered a noteworthy correlation between the initial medications prescribed for preventing thromboembolic events in nonvalvular atrial fibrillation. Our findings indicate that NOACs were predominantly prescribed by the medicine department (77.1%), while warfarin was also predominantly prescribed by the medicine department (58.5%) with a statistically significant difference (*p* < 0.001). This outcome is not unexpected, as physicians are primarily responsible for managing cases of thromboembolic events and atrial fibrillation.

NOACs were predominantly prescribed by the medicine department (63.2%) as the primary line of medication for preventing thromboembolic events in patients with atrial fibrillation resulting from moderate or severe mitral stenosis caused by rheumatic heart disease. Warfarin (71.9%) was also prescribed by the medicine department for the same purpose. The medicine department's preference for prescribing warfarin over NOACs is mostly influenced by comorbidities that may occasionally prevent the commencement of therapy that adheres to guidelines, as indicated by the research conducted by Farinha et al. [7] These findings regarding the prescription of DOAC over VKA in mitral stenosis associated atrial fibrillation is in line the recent trial conducted by Ghamrawy et al. where they found that among patients with mitral stenosis related atrial fibrillation VKA therapy led to a lower rates of cardiovascular events or death than DOAC (Rivaroxaban) therapy [8].

Cost is a significant consideration, with 73.2% of doctors identifying the price of DOACs as a significant obstacle to their use. This figure is comprehensible given the prevalent low socioeconomic level of most Sudanese individuals. Another prevalent practice is for doctors to make assumptions about their patients' poverty and give them the most affordable and readily accessible drugs, such as Warfarin.

### 4.1. Limitations

Our study may be limited by the relatively small samples size (144) as it could not be enough to provide a strong conclusion about the barriers that interferes with the wide usage of NOACs. Also the unequal representation of different departments could serve as a selection bias as different departments may have different views about the barriers. The ongoing armed-conflict in Sudan which stated at 15 April 2023 has prevented us from going further with the research and reach a larger sample size.

## 5. Conclusion

The study examined the obstacles that hinder the widespread use of NOACs for the prevention of thromboembolic events among clinicians in Sudan. The primary obstacle to prescribing NOACs is the absence of a dedicated antidote and the absence of a multidisciplinary approach to their prescription. Another significant barrier is the limited understanding of international guidelines, with many participants preferring to seek specialist advice. Personal experience with the efficacy of NOACs also contributes to a noteworthy proportion of the barriers. Additionally, the high cost of NOACs is a considerable factor. Physicians appear to possess the highest level of expertise among all specialties in the survey.

## 6. Recommendations

• Implementing a multidisciplinary team for the prescription of DOACs and ensuring that clinicians adhere to updated recommendations.
• Introduce the DOACs and include them in the government's insurance plan.
• Additional research should be conducted to examine the specific obstacles within each group of doctors, with particular attention given to the orthopedic department.

## Figures and Tables

**Table 1:** Departmental distribution among research participants.

Department	Frequency	Percent (%)
ER department	19	13.2
Medicine department	66	45.8
Other departments	27	18.8
General surgery department	8	5.6
Cardiothoracic surgery	6	4.2
Orthopedic surgery	18	12.5
Total	144	100

**Table 2:** Demonstrates the different medical degrees.

Medical degree	Frequency	Percent (%)
Consultant	17	11.8
House officer	22	15.3
General practitioner	36	25.0
Registrar	36	25.0
Specialist	33	22.9
Total	144	100

**Table 3:** Reflect the years of experience.

Years of experience	Frequency	Percent (%)
11–20 years	18	12.5
21–30 years	19	13.2
5–10 years	6	4.2
Less than 5 years	22	15.3
More than 30 years	74	51.4
Total	144	100

**Table 4:** Awareness regarding 2021 guidelines about DOAC usage.

Answer	Frequency	Percent (%)
No	73	50.7
Yes	71	49.3
Total	144	100

**Table 5:** Association between types of drugs prescribed for thromboembolism prevention and departments.

			Department						
			ER	Medicine	Others	General surgery	Cardiothoracic surgery	Orthopedic surgery	
First line for prevention of thromboembolic events		Count	1	60	22	8	4	0	
			1.1%	63.2%	23.2%	8.4%	4.2%	0.0%	
	DOACs	Count	4	2	2	0	2	0	
			40.0%	20.0%	20.0%	0.0%	20.0%	0.0%	
	Heparin LMW/UFH	Count	13	3	2	0	0	17	
			37.1%	8.6%	5.7%	0.0%	0.0%	48.6%	
	Warfarin	Count	1	1	1	0	0	1	
			25.0%	25.0%	25.0%	0.0%	0.0%	25.0%	

Total		Count	19	66	27	8	6	18	
			13.2%	45.8%	18.8%	5.6%	4.2%	12.5%	< 0.001^∗^

^∗^ *p* value < 0.001 indicates a significant association.

**Table 6:** Association between first line of drugs prescribed for thromboembolism prevention and departments.

			Department						Total
			ER	Medicine	Others	General surgery	Cardiothoracic surgery	Orthopedic surgery	
First line medication for the prevention of thromboembolic events nonvalvular atrial fibrillation		Count	15	2	16	0	4	15	
			28.8%	3.8%	30.8%	0.0%	7.7%	28.8%	
	DOACs	Count	1	37	5	2	1	2	
			2.1%	77.1%	10.4%	4.2%	2.1%	4.2%	
	DOACs/warfarin	Count	0	1	0	0	0	0	
			0.0%	100.0%	0.0%	0.0%	0.0%	0.0%	
	NOACs/DOACs	Count	0	2	0	0	0	0	
			0.0%	100.0%	0.0%	0.0%	0.0%	0.0%	
	Warfarin	Count	3	24	6	6	1	1	
			7.3%	58.5%	14.6%	14.6%	2.4%	2.4%	

Total		Count	19	66	27	8	6	18	
			13.2%	45.8%	18.8%	5.6%	4.2%	12.5%	< 0.001^∗^

^∗^ *p* value < 0.001 indicates a significant association.

**Table 7:** Association between the type of drug and department for using it as a first line medication for the prevention of thromboembolic events in patients with atrial fibrillation due to mitral stenosis.

			Department						
			ER	Medicine	Others	General surgery	Cardiothoracic surgery	Orthopedic surgery	
First line medication for the prevention of thromboembolic events in patients with atrial fibrillation due to moderate to severe mitral stenosis caused by rheumatic heart disease		Count	14	5	17	0	5	17	
			24.1%	8.6%	29.3%	0.0%	8.6%	29.3%	
	DOACs	Count	2	12	4	0	0	1	
			10.5%	63.2%	21.1%	0.0%	0.0%	5.3%	
	DOACs/warfarin	Count	0	2	0	0	0	0	
			0.0%	100.0%	0.0%	0.0%	0.0%	0.0%	
	NOACs/DOACs	Count	0	1	0	0	0	0	
			0.0%	100.0%	0.0%	0.0%	0.0%	0.0%	
	Warfarin	Count	3	46	6	8	1	0	
			4.7%	71.9%	9.4%	12.5%	1.6%	0.0%	

Total		Count	19	66	27	8	6	18	
			13.2%	45.8%	18.8%	5.6%	4.2%	12.5%	< 0.001^∗^

^∗^ *p* value < 0.001 indicates a significant association.

**Table 8:** Correlation between different research questions.

		Medical degree	Years of experience	Barriers related to doctors	Barriers of patient doctor relationship	Thoughts related to the drug	Barriers related to resources	Reporting of outcomes	Direct oral anticoagulant prescription
Medical degree		1.000	0.650^∗∗^	−0.219^∗∗^	0.031	−0.211	−0.105	−0.017	0.371^∗∗^
			< 0.001	0.008	0.716	0.011	0.209	0.837	< 0.001
	*N*		126	144	144	144	144	144	123

Years of experience			1.000	−0.194	0.166	−0.071	−0.096	0.030	0.303^∗∗^
				0.029	0.063	0.427	0.287	0.739	< 0.001
	*N*			126	126	126	126	126	123

Barriers related to doctors				1.000	0.105	0.341^∗∗^	0.366^∗∗^	0.195	−0.266^∗∗^
					0.212	< 0.001	< 0.001	0.019	0.003
	*N*				144	144	144	144	123

Barriers related to patient doctor relationship					1.000	0.393^∗∗^	0.144	0.176	0.063
						< 0.001	0.084	0.035	0.486
	*N*					144	144	144	123

Barriers of thoughts related to the drug						1.000	**0.284** ^∗∗^	**0.166**	**−0.229**
							< 0.001	0.046	0.011
	*N*						144	144	123

Barriers related to resources							1.000	**0.248** ^∗∗^	−0.131
							.	0.003	0.150
	*N*							144	123

Reporting of outcomes as a barrier for DOACs prescription.								1.000	0.003
									0.972
	*N*								123

*Note:* The bold values with *p* < 0.05 is considered statistically significant.

^∗∗^Indicate variables with significant association with the presence of barriers to NOAC use.

## Data Availability

All data generated or analyzed during this study are included in this published article.
